# A Review of CFD Modelling and Performance Metrics for Osmotic Membrane Processes

**DOI:** 10.3390/membranes10100285

**Published:** 2020-10-15

**Authors:** Kang Yang Toh, Yong Yeow Liang, Woei Jye Lau, Gustavo A. Fimbres Weihs

**Affiliations:** 1 Faculty of Chemical and Process Engineering Technology, College of Engineering Technology, Universiti Malaysia Pahang, Lebuhraya Tun Razak, Kuantan 26300, Pahang, Malaysia; mkc18013@stdmail.ump.edu.my; 2 Centre of Excellence for Advanced Research in Fluid Flow (CARIFF), Universiti Malaysia Pahang, Lebuhraya Tun Razak, Kuantan 26300, Pahang, Malaysia; 3 Advanced Membrane Technology Research Center, School of Chemical and Energy Engineering, Universiti Teknologi Malaysia, Skudai 81310, Johor, Malaysia; lwoeijye@utm.my; 4 School of Chemical and Biomolecular Engineering, The University of Sydney, Sydney, NSW 2006, Australia; gustavo.fimbresweihs@sydney.edu.au

**Keywords:** CFD, spiral wound membrane, performance metrics, desalination, modelling

## Abstract

Simulation via Computational Fluid Dynamics (CFD) offers a convenient way for visualising hydrodynamics and mass transport in spacer-filled membrane channels, facilitating further developments in spiral wound membrane (SWM) modules for desalination processes. This paper provides a review on the use of CFD modelling for the development of novel spacers used in the SWM modules for three types of osmotic membrane processes: reverse osmosis (RO), forward osmosis (FO) and pressure retarded osmosis (PRO). Currently, the modelling of mass transfer and fouling for complex spacer geometries is still limited. Compared with RO, CFD modelling for PRO is very rare owing to the relative infancy of this osmotically driven membrane process. Despite the rising popularity of multi-scale modelling of osmotic membrane processes, CFD can only be used for predicting process performance in the absence of fouling. This paper also reviews the most common metrics used for evaluating membrane module performance at the small and large scales.

## 1. Introduction

**Figure 1:**
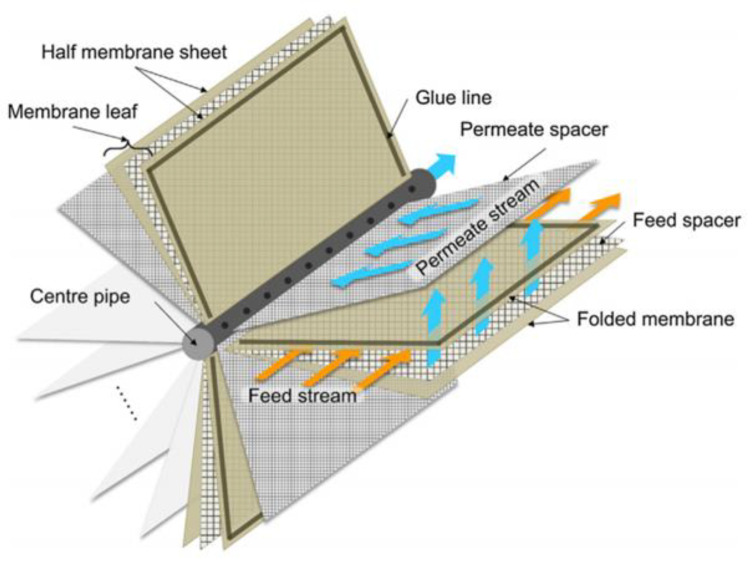
Schematic diagram of a spiral wound membrane (SWM) module [2].

Membrane-based desalination processes have gained global attention due to their simplicity and lower operating cost compared with thermal-based desalination processes [1]. For the desalination using reverse osmosis (RO) membranes, spiral wound membrane (SWM) modules are the standard configuration. In this module design (as shown in Figure 1), two flat sheet thin film composite (TFC) RO membranes (labelled as “membrane leaf”) are sealed together on three sides (forming a type of envelope sheet), with the membrane porous layers (a.k.a. substrate) facing each other and a permeate collection material placed between them. Feed spacers are then placed on top of the selective layer of each sheet, and this is followed by rolling the sandwiched sheets into a spiral format around a perforated central tube to complete the module fabrication. 

SWM modules have been used for industrial applications of seawater RO (SWRO) since the 1960s [3], and their use has been further extended to nanofiltration (NF) in the early 1980s [4]. For emerging osmotic membrane processes such as forward osmosis (FO) and pressure retarded osmosis (PRO), the development of SWMs is still at an early stage and is only limited to lab-scale studies [5,6,7,8]. Although some researchers report that the hollow fibre membrane configuration is more suitable for FO and PRO processes due to its higher packing density, self-mechanical support properties and better flow control on both sides of the membrane (i.e., lumen and outer surface of fibre) [9,10], a large number of studies published over the years have used flat sheet membranes for assessing FO and PRO processes [6,11,12,13,14,15,16,17].

Of the osmotic processes, RO has been the focus of much attention for six decades [18]. More than 50% of the desalination plants installed in the world are based on RO technology [1,19]. RO is a pressure-driven process which applies hydraulic pressure on a feed stream containing high solute concentration, forcing clean water with much lower solute concentration (in most of the cases, less than 150 mg/L total dissolved solids, TDS) to diffuse through a semi-permeable membrane. The RO process is similar to other pressure-driven processes such as NF, with the main differences being in terms of membrane pore size and the ability to reject molecular or ionic species [20,21]. For example, RO membranes exhibit monovalent salt rejection (e.g., NaCl) above 95%, while the larger pore sizes of NF membranes are only able to achieve between 30% and 90% rejection of that salt.

Nonetheless, RO is an energy-intensive process and reduction of energy consumption remains one of the main research priorities to date [22,23,24,25]. Numerous studies have been conducted to attempt to reduce the energy consumption of RO processes, and they can be classified into: (1) developing ultra-high permeance membrane using advanced materials, e.g., graphene oxide (GO) membranes [26,27]; (2) using highly efficient energy recovery devices [28]; (3) using optimisation-based control systems [29,30,31]; and (4) optimising design of RO systems [32,33]. Despite these efforts, advances in reduction of energy consumption have almost plateaued. For example, improvements in membrane permeability have only led to an asymptotic change in flux and energy consumption due to the high concentration polarisation that occurs at higher fluxes [34,35]. This has prompted the study of different types of osmotic membrane processes, e.g., FO and PRO, in an effort to further minimise energy consumption.

In contrast to the case of RO, permeate flux in FO does not rely on transmembrane pressure as its driving force. Rather, permeation across the membrane in FO is driven by the solute concentration difference (or osmotic pressure gradient) between the draw and feed solutions. Moreover, in pressure assisted osmosis (PAO), which is a variation of FO, permeate flux is driven by the combined hydraulic force and osmotic gradient [36]. FO processes have gained attention due to their advantages over RO in terms of lower energy consumption, lower membrane fouling propensity and considerably higher water recovery [37,38]. However, FO cannot be considered a replacement technology for RO, as solvent flux occurs from the dilute to the concentrated solution, in effect “diluting” the draw solution, as opposed to RO where the high concentration feed is further concentrated to produce a more pure solvent in the permeate stream. Nevertheless, FO process shows much greater potential in handling feed solution composed of high ion/foulant concentration in comparison to the RO process due to the absence of hydraulic pressure. This results in lower degree of FO membrane surface fouling even when it is used to treat highly concentrated solution.

**Figure 2:**
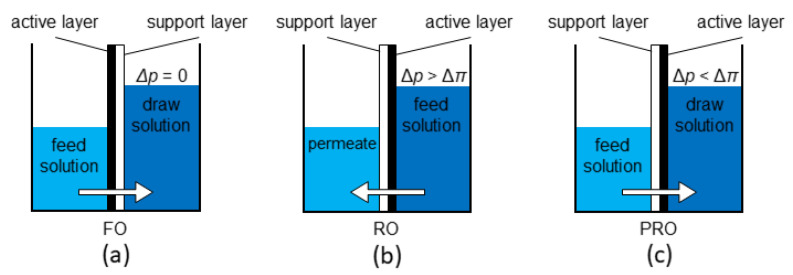
Schematic figures illustrating the principles of (**a**) forward osmosis (FO), (**b**) reverse osmosis (RO) and (**c**) pressure retarded osmosis (PRO).

The PRO process has a similar configuration to that of FO, except that the selective side of the membrane faces a higher concentration (draw) solution [39,40,41]. In PRO, the solution in the draw side is partially pressurised and kept below its osmotic pressure in order to create a net osmotic driving force for water to flow from the feed side to the draw side. It is worth noting that PRO is the opposite process of RO. RO utilises hydraulic energy to exceed the osmotic pressure and produce fresh water, whereas PRO utilises the osmotic potential energy arising from the salinity gradient to generate a type of renewable energy, where the permeate is depressurised for energy generation purposes. Figure 2 compares the principles of operation for the FO, RO and PRO processes.

Membrane processes have traditionally been analysed using mathematical modelling. This allows researchers to describe and analyse real membrane problems, either from first principles or black-box modelling approaches. There are several benefits when employing a mathematical modelling approach for the analysis of the performance of RO, FO and PRO membranes [42,43]. These include better prediction of the membrane performance for a range of membrane properties and operating conditions, and deeper understanding of the effects of flow dynamics and water transport mechanisms. Thus, mathematical modelling is an important element which can complement experimental studies in designing optimal operating conditions or configurations [44,45].

In recent years Ahmed et al. [45] reviewed the advances in modelling and optimisation for desalination in the context of transport phenomena, energy usage, fouling and hybrid desalination technologies. However, their review only considered simple transport phenomena without taking into account the current advent of computational fluid dynamics (CFD) and multi-scale modelling techniques for membrane processes. As such, addressing these gaps is the main focus of this review. CFD is a powerful tool for visualising hydrodynamics, mass transfer and fouling phenomena inside spacer-filled membrane channels, such as those encountered in SWM modules. Although the length scales usually considered in CFD models are of the order of millimetres, these small-scale results can be extrapolated to make full large-scale predictions of module-scale performance [33,46].

In addition, the accurate choice of model performance indicators or metrics is another crucial test when evaluating the effectiveness of proposed process modifications, such as novel spacer geometries, optimal operating conditions, as well as module configurations. Many of the currently used performance metrics are expressed in the form of dimensionless unit to ensure they are independent of the dimensions of the membrane system [47,48]. The dimensionless nature of these metrics is necessary to allow the comparison of data from different studies and to facilitate membrane scale-up. However, to our best knowledge, there is no thorough review on how to select the best module performance indicators for desalination. Hence, this review also aims to summarise and compare some proposed performance metrics, for both small-scale (i.e., CFD scale) and large-scale membrane processes.

This review first outlines recent progress in spacer design for SWM modules, followed by a description of the existing and main challenges in modelling membrane systems. For the sake of conciseness, we only review how CFD and multi-scale models can be employed to gain insights into the flow enhancement mechanisms for osmotic membrane processes without listing all the assumptions and mathematical equations. The review then discusses and compares module performance metrics in the context of the development of comprehensive and precise assessments of SWM modules for RO, FO and PRO processes.

## 2. Development of New Spacer Designs

SWMs consist of a sandwich of flat sheet membranes, spacer filaments and porous permeate flow material, wrapped around a central permeate collecting tube [49]. The presence of spacers in the feed channels of the SWM helps improve permeate flux by promoting fluid mixing that enhances mass transfer and reduces concentration polarisation [50,51,52], albeit at the cost of an increased pressure loss [53,54,55]. As spacer geometry has a large impact on the flow distribution (and hence on mass transfer), spacer modification remains an active and ongoing research field aiming to improve on three important issues: (1) flux enhancement [52,56,57], (2) pressure loss reduction [52,55,56,58], and (3) mitigating the fouling caused by the spacer whereby some regions experience low levels of fluid mixing [52,55,56]. This section reviews some of the latest proposed novel spacer designs and compares their performance for desalination.

**Figure 3:**
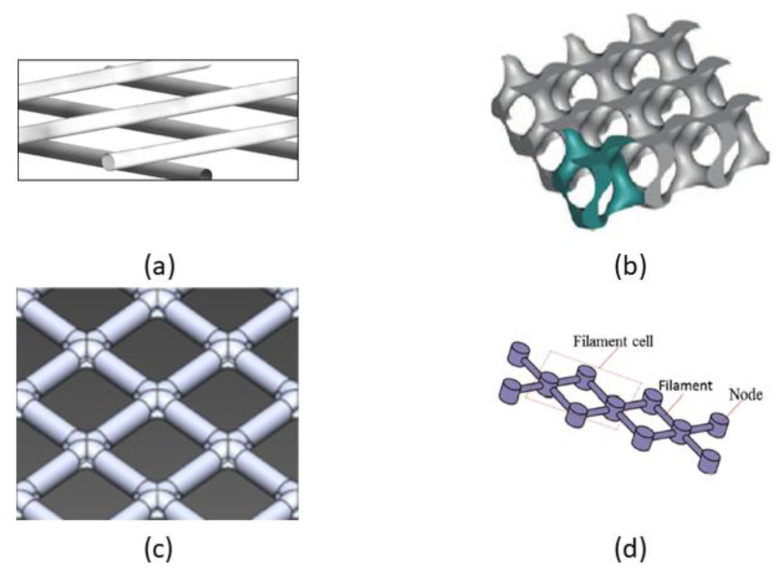
Spacer geometry configurations for (**a**) conventional spacer [68], (**b**) triply periodic minimal surface (TPMS) spacer [52], (**c**) perforated spacer [55] and (**d**) submerged spacer with nodes [58].

The most commonly used spacer geometries in SWM modules comprise two layers of non-woven spacer filaments (Figure 3a) with differing cross section shapes, sizes and flow attack angles [51,59,60]. Given the current advances in CFD and 3D printing technologies, many hurdles in the design and manufacture of novel spacers with complicated configurations have been overcomed. Nonetheless, 3D printing technologies of spacer are still relatively less explored by researchers, though current technologies (viz. Additive Manufacturing, AM [61]) show potential in manufacturing small scale prototypes in sizes of the order of microns. There is a significant research gap to produce a more intricate spacer prototypes using 3D printing technologies. Furthermore, such intricate prototypes are generally not able to be manufactured in large scale using traditional spacer manufacturing technique. This is because 3D printing of spacer meshes requires a very high printing resolution (normally of the order of hundreds of microns) [61] and would result in high manufacturing costs for industrial applications [62]. In view of this, CFD modelling is often preferred over experimental tests for predicting the performance of novel spacer geometric designs. Hence, numerical studies of several complex spacer geometries can be found in the literature, such as the triply periodic minimal surface (TPMS) structure (Figure 3b) [52,60,63,64,65], perforated spacer geometries (Figure 3c) [55] and submerged spacers with column nodes (Figure 3d) [56,58,66]. Although a previous review [18] has discussed the criteria for optimising spacer design, most designs are still restricted to simple cylindrical or two-layer spacer geometries. Table 1 summarises some of the most important CFD and experimental works related to novel spacer development. 

The TPMS spacers make use of fluid dynamics principles by interlinking the flow channels to reduce restrictions to axial feed flow [52,60,63,65]. The lack of sharp edges in the TPMS spacer also minimises pressure drop through gradual enlargement or contraction of the flow path [67]. This novel spacer design was found to outperform the RO water flux induced by commercial feed spacers in brackish water by 15.5% and reduce pressure drop by more than 5% [52]. In addition, its proponents claimed that the TPMS design is capable of reducing biofouling by at least 45%.

Another strategy to improve spacer geometry is by perforating the spacer filaments [55], as this has the potential to enhance permeate flux and minimise pressure drop. This strategy clearly highlights that proper modification of spacer geometry has direct effects on the hydrodynamics of the feed channel, reducing not only pressure loss but also enhancing mass transfer. In another study, Ali et al. [58] found that a submerged design with column nodes has the potential to cut pressure drop to a third of that of a spacer with thicker filaments (i.e., larger diameter). However, the base case selected in that study is not a typical two-layer spacer geometry. Hence, it is unclear how the proposed novel submerged geometry with column nodes performs when compared with a conventional spacer geometry.

Although several novel spacer geometries have been proposed, none of the previously mentioned studies have performed a CFD analysis of mass transfer phenomena, and only some of them included a numerical analysis of the hydrodynamics in the spacer-filled channels [55,58]. This may be related to the high mesh refinement requirement near the surface of the intricate or sharp features of the proposed novel spacers, so as to precisely capture the hydrodynamics and mass transfer near the spacer and membrane surfaces. Liang et al. [46] highlighted the importance of a mesh refinement study for modelling complex geometries with intricate features, i.e., multi-layer spacers. For example, at least 20 cells within the boundary layer in the vicinity of the spacer and membrane surfaces are required, in order to accurately resolve the wall shear and mass transfer coefficient, and to obtain grid convergence indices (GCI) of below 2.5%.

## 3. Existing CFD Models and Their Challenges 

### 3.1. Reverse Osmosis (RO) Modelling

Several CFD software packages are available and commonly used for CFD modelling of membrane channels in the literature, including commercial CFD suites such as ANSYS-CFX, ANSYS-FLUENT, COMSOL Multiphysics, as well as open source software such as OpenFOAM. Since several exhaustive reviews focusing on CFD modelling of spacer-filled channels are documented in the literature [69,70], this section only discusses some of the recommendations proposed in recent years (2015 to date) and evaluates their relevance. From the previous relevant reviews, several recommendations are proposed for future work. These include: (1) coupling mass transfer with fouling models [69], (2) validating CFD through experimental work, such as particle image velocimetry (PIV) for flow visualisation [69], and (3) developing robust and reliable SWM module-scale models [70]. 

For simplicity of computation, CFD modelling of RO processes usually focuses on fluid flow modelling on the feed side without taking into consideration the permeate channel. This is because there is a very small pressure drop on the permeate channel and concentration polarisation (CP) on the permeate side is negligible. The permeate pressure at any point within the SWM module can be assumed at about 1 bar. For these reasons, only the water flux and solute rejection that take place on the feed side of the membrane are investigated in CFD modelling of RO.

The reliability and applicability of CFD as a prediction tool in terms of feed conditions (e.g., seawater salinity and feed flow rate) have been well discussed and validated in the literature [71,72]. For example, the reliability of CFD simulations is not greatly affected by the inclusion of density variations, as buoyancy effects do not dominate [73]. Similarly, viscosity does not play a big role in the main mass transfer mechanisms that drive concentration polarisation and performance decrease due to that phenomenon [71]. Nonetheless, care must be taken when modelling high salinity conditions, as the membrane surface concentration may increase above the saturation limit for salt precipitation, in which case scaling modelling should be included.

At the time of writing, CFD studies of RO processes have mainly focused on either 2D unsteady state [74,75,76,77,78,79] or 3D steady state [51,54,57,66,80,81,82]. It is worth noting that the ability of 2D and 3D CFD modelling for predicting mass transport and concentration polarisation has been well established in the literature [46,68,74,83]. While 3D unsteady models would provide more insights than 2D unsteady or 3D steady studies, they require very significant computational power and time to converge. To the best of our knowledge, the works conducted by Kerdi et al. [55] and Koutsou et al. [84] are the only studies that simulated 3D unsteady flow in spacer-filled channels. However, these works are limited to analysing hydrodynamics, without including mass transfer. This is because mass transfer would require computational resources several times larger than hydrodynamic-only studies.

Another important growing trend in modelling RO membrane channels is the emergence of different types of fouling models [79,85,86,87,88,89]. Of the different types of fouling, biofouling modelling perhaps is gaining the most attention whether in the past or present [86,88,90,91] as it is the type of fouling that has the largest effect on RO membrane performance due to excessive accumulation of biomass on the membrane surface. Nevertheless, developing a good biofouling model requires information of biofilm properties, which can only be obtained through experimental studies [86]. Biofilm mechanical properties, for instance, can be obtained from a combination of numerical modelling and data obtained from optical coherence tomography (OCT) scans [92]. Most 2D and 3D CFD studies of biofouling have been validated experimentally [93,94,95], and this gives confidence in the prediction of biofouling on the membrane surface. However, one of the main issues associated with this kind of modelling is that it requires a significant amount of computational resources, and often necessitates the integration of a collection of software packages due to the very different length scales involved: unsteady hydrodynamics requires time steps of the order of microseconds, whereas fouling in the feed channel occurs over long time scales of the order of days or weeks.

Besides biofouling in actual RO practice, other types of fouling such as scaling, particulate and organic fouling can occur simultaneously and interact with each other [96]. To our best knowledge, currently only one work conducted by Radu et al. [97] has numerically modelled the interaction between scaling and biofouling. It must be emphasised that modelling the interaction between different types of fouling, and the effects of the interaction between hydrodynamics and mass transfer on fouling is very challenging, mainly due to the lack of understanding and knowledge of the first principles associated with those interactions. More efforts towards these research directions are therefore required. 

A review by Bucs et al. [86] highlighted that a complete computational technique should take into account not only the unsteady flow but also the fluid-biofilm interaction, in order to investigate both permeation and biofilm removal. In addition, the authors proposed a possible optimisation loop which used X-ray computed tomography (CT scanning step) to obtain a more accurate spacer geometry measurement for computer aided design (CAD) and CFD simulation analysis. They claimed that X-ray computed tomography is useful in resolving the over-simplified spacer geometry in CFD simulations [98,99]. This is because real spacer meshes have some deformed features due to the polymer extrusion fabrication technique [98]. On the other hand, Horstmeyer et al. [99] found that a cylindrical spacer structure overestimates CP when compared with the geometries measured by CT scans. This is because the flow distribution and shear stress are incorrectly predicted when the narrow flow sections formed between spacer filaments and membrane are not taken into consideration.

Although the most common water sources considered as feed for RO systems (such as seawater and brackish water) contain multiple ionic solutes, previous modelling studies have assumed a single type of solute in the system. To the best of our knowledge, no study has been carried out so far to evaluate the effects of interactions between different solutes on the membrane performance. This can be due to the lack of numerical tools for implementing them in CFD. Under this circumstance, the mass transport equations become extremely stiff when electric field effects related to the solute charges are included. The modelling of multiple ionic solvents has only been performed for electrodialysis studies, and it was limited to 1D studies [100]. Modelling in 2D or 3D results in a high degree of stiffness in the mass transport equations, making the diffusion term non-homogenous if simple CFD techniques are used. Therefore, there are opportunities for future CFD work which can take into account multiple solute interactions in RO systems, as well as other osmotic driven processes (FO, PRO).

**Figure 4:**
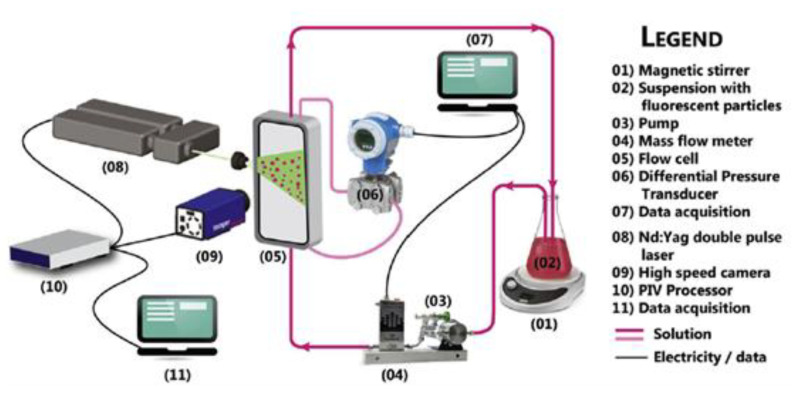
Experimental setup for flow visualisation of PIV [59].

**Figure 5:**
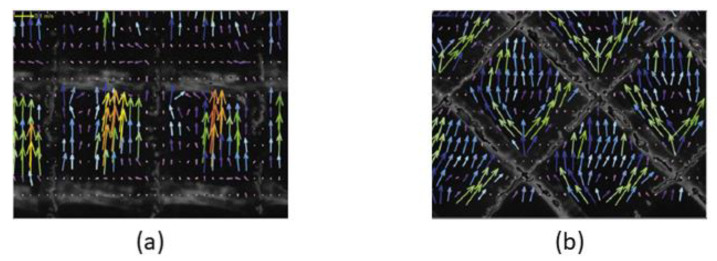
Velocity vector obtained from particle image velocimetry (PIV) imaging at centre of membrane channel for (**a**) ladder orientation and (**b**) normal orientation [59].

A commonly raised issue regarding CFD studies is the difficulty of obtaining data suitable for validation. In order to verify and validate CFD results, PIV provides a direct and high-resolution technique to visualise flow [53,59,101]. However, it is important to note that the density of particles that are tracked with the laser needs to approximate that in the fluid in order to accurately predict the flow behaviour. This is because the particle movement will be affected by buoyant forces if their density is not equal to that of the fluid. In addition, flow field measurements can also be affected if there is a significant change in density due to sharp changes in concentration, or by shear forces such as shear-induced diffusion. Another major limitation of this technique is the requirement of a very high resolution camera in order to capture time- and spatially varying flow velocity [102]. Although some progress has been made in using PIV for studying membrane channels, only a handful of studies are available in the literature [53,59,101,102,103]. Figure 4 shows the PIV experimental setup reported in the work of Haidari et al. [59]. That setup was developed to measure pressure loss and visualise the temporal and spatial velocity variations inside a spacer-filled channel. Examples of velocity vector fields at the centre of membrane channel for different orientations are shown in Figure 5. However, the authors of that work [59] acknowledge that further study is required to evaluate the effect of *z*-velocity on the *u-* and *v*-velocities.

Several novel spacers have been proposed in the literature [52,55,56,57,63], but those studies were performed at lab scale experiment (i.e., test section of order of centimetre) or small-scale simulation (i.e., a few unit cells in millimetre-scale). Real RO membrane desalination systems are in fact in several meters in length [46,104,105]. Thus, multi-scale models are required to predict the effectiveness of a novel spacer in full-length SWM desalination systems [46,105,106,107]. These models refer to the combination of the small-scale details of the spacer geometry with the large-scale RO module. More specifically, small-scale CFD simulations can be used to generate correlations for predicting the Sherwood number and friction factor dependence on Reynolds number. These predictions can be subsequently used to determine the mass transfer coefficient and pressure drop, which are then used as inputs to solve ordinary differential equations (ODEs) for flux, salt and pressure drop along the membrane module in a large-scale module [46]. Several small-scale CFD studies have included fouling effects during analysis [79,87], but until now robust multi-scale models incorporating fouling phenomena are yet to be developed. Future work on this is thus highly recommended for more accurate prediction of long-term membrane performance. 

### 3.2. Forward Osmosis (FO) and Pressure Retarded Osmosis (PRO) Modelling

**Figure 6:**
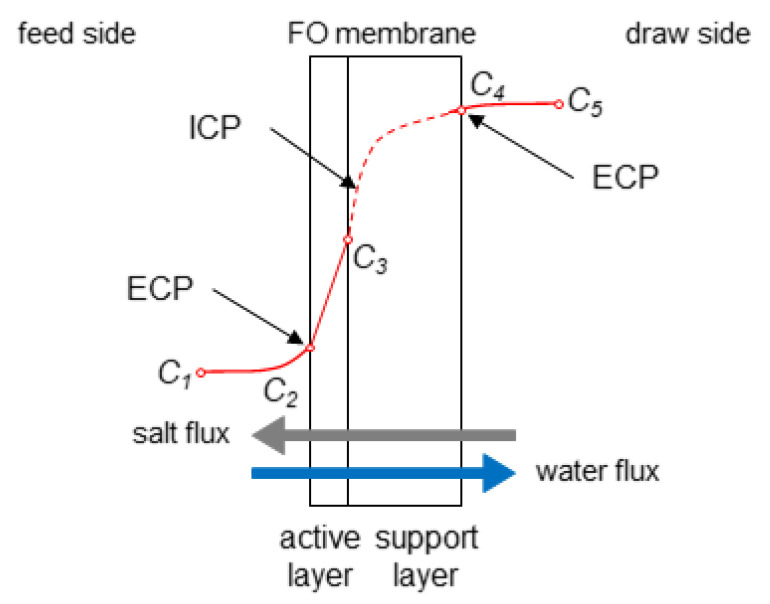
Concentration profile across an FO membrane showing the different types of concentration polarisation.

Recently, there has been significant research focus on the FO process due to its low operational energy consumption [40] and lower propensity to fouling [108]. Table 2 summarises recent findings through CFD modelling of the FO process. Compared with the RO process, FO performance is more sensitive to membrane characteristics because FO is purely driven by the osmotic pressure difference; thus, flux enhancement largely depends on membrane properties. In addition to the hydrophilicity and pore size of the skin layer, the performance of FO also depends on the substrate morphology including thickness, porosity and tortuosity [109], as well as the solute concentration difference across the membrane [110,111,112]. Therefore, two important mechanisms for flux enhancement in FO are: (1) increasing water permeability and (2) improving mixing on both draw and feed channels to minimise CP. Unlike in CFD modelling of RO, both sides of the FO membrane need to be modelled. This is because the solute build-up for FO not only occurs in both membrane channels (feed and draw), but also within the porous layer of the membrane [113]. As shown in Figure 6, there are two types of external concentration polarisation (ECP), where the difference between *C_1_* and *C_2_* gives rise to the feed ECP, and the difference between *C_4_* and *C_5_* gives rise to the draw ECP. The difference between *C_3_* and *C_4_*, on the other hand, gives rise to the internal concentration polarisation (ICP) (which for FO is on the draw side, but for PRO is on the feed side).

Most importantly, it has been reported that modelling that does not consider ICP could overestimate water flux (by up to 15%), especially when the draw solution concentration is high (2 M NaCl) [114]. Nevertheless, Sagiv et al. [115] reported that the resistance to water permeation due to ICP in FO is smaller than that for ECP, as it accounts for less than 2.5% of the overall resistance to water permeation. Thus, the authors suggested that further research should focus on reducing ECP rather than ICP.

A general boundary condition for the solute mass fraction that must be applied at both sides of the membrane can be described as follows:

(1) $$\rho w_{m}J_{w}-\rho D\;{\left(\nabla w\right)}_{m}=J_{s}$$

where *ρ*, *D* and *w* denote fluid density, solute diffusivity and solute mass fraction, and the *m* subscript denotes that the concentration values are taken at the membrane surface (either feed or draw side, respectively). It is important to note that *J_s_* can only be determined when experimental values for the solute (*A*) and membrane (*B*) permeability parameters are available [116]. 

With respect to computational performance, the simulations results reported by Gruber et al. [116] demonstrate that a mesh with the order of 100 cells perpendicular to membrane in both feed and draw channel for 2D rectangular empty channel is sufficiently validated with the flux within 10% error, and only took about 1 h to converge to steady-state. Nevertheless, this study is limited to a simple 2D analysis. As the Reynolds number inside the FO membrane channel is typically of the order of 100 [117], the steady or transient flow can be solved efficiently by directly solving the transport equations without using a turbulence model, and using fine grid elements, especially near the membrane surface. 

Some of the strategies to reduce ICP and ECP for FO are placing spacers far from the membrane surface on the feed side and/or placing spacers near the membrane in draw side [11]. By employing this approach, the membrane water flux could be enhanced by 8–19% [11]. Similar to what happens in the RO process, the presence of spacers could also promote scaling near the surface of the FO membrane [7]. The presence of spacer filaments tends to create recirculating zones downstream of the flow obstructions, promoting crystallisation and growth of gypsum scales. This, as a result, significantly deteriorates the water flux through the membrane. However, it must be pointed out that very few studies have modelled fouling for FO, owing to the difficulties to modelling the interaction between foulants in both the feed and draw sides of the membrane. 

Another important observation is that most CFD studies of FO systems are restricted to only 2D analyses of spacer design [41,109,115,116,118], except few employing 3D simulations [36,119]. Hence, more research in these directions is required to shed insights into the development of improved spacers suited for FO. Another major challenge faced by CFD modelling of the FO process is the analysis of the large-scale FO module design. Even though CFD analysis provides some interesting insights into the flow patterns for FO process, it cannot be used to predict effectiveness of overall module-scale FO. In order to capture the fundamentals of CP for FO, Jeon et al. [111] proposed a module-scale model that combines both fundamental and empirical models to predict the performance of FO in SWM module. The authors underlined that the prediction error in spiral wound performance is caused by transmembrane pressure in which an increasing transmembrane pressure may shrink the draw channel height, leading to higher draw side flow rate and water flux. Thus, including transmembrane pressure in the fitting process is crucial to enhance the accuracy of module-scale predictions. However, the authors acknowledged the limitations of their model because of the exclusion of fouling effect and only applicable to a single SWM module.

Unlike RO and FO, CFD modelling of PRO has received very little attention from the membrane scientific community. The first numerical study of PRO was published by Straub et al. [120] using a 1D numerical model that aimed to solve the mass transfer equation at the module-scale by taking into account the ICP, ECP and reverse salt flux (RSF). The study focused on maximising specific energy and power density extraction by manipulating applied hydraulic pressure, initial feed flow rate fraction and membrane area. However, their study found that a high specific energy tended to produce very low power density, implying a challenge for PRO module-scale operation. The researchers also observed that the river water-seawater pairing used in the study resulted in comparably low power generation. Nevertheless, if the river water-seawater pairing is replaced with wastewater-desalination brine, a 2-fold increase in the extractable specific energy could be expected because of the much higher osmotic gradient between two solutions. In another module-scale analysis carried out by Soltani and Struchtrup [121], it was found that a dual-stage system showed improvement in comparison to a single-stage system in terms of specific energy generation. 

To the best of our knowledge, there is only one study that has modelled the PRO process, which was based on OpenFOAM [43]. The researchers used a 2D spacer-free (empty) channel, which extended a commonly used semi-analytical model into a 2D CFD model and employed both semi-analytic and CFD modelling to investigate the effect of hydraulic pressure, cross flow velocity and membrane properties on the water flux in a PRO process. Their results showed that CFD modelling enhanced the prediction accuracy compared to the commonly used semi-analytic models, especially in the case where improved membrane properties (i.e., higher permeability and selectivity) were considered. However, a direct validation between PRO CFD model and experimental data was not carried out in this work and only some verification of numerical results against RO and FO CFD model were reported. 

Table 3 summarises the literature on modelling studies for PRO systems. Although some module-scale analyses are available [120,121], detailed 3D CFD modelling for the PRO process has yet to be investigated. The insight of 3D modelling is important for scaling up the PRO processes; thus, further modelling work for PRO is recommended. 

Table 4 summarises the boundary conditions used in CFD models for channels with or without spacers in RO, FO and PRO process. As can be seen in Table 4, many boundary conditions used in RO are also applicable for FO and PRO, even for 3D studies. In particular, the inlet, outlet, wall and side opening boundary conditions are applicable for all osmotic membrane processes. The differences in boundary conditions are mostly centred on the membrane boundary condition, which include different terms depending on the process. For example, for RO it is typical to use the solute intrinsic rejection of the membrane on the concentration boundary condition. On the other hand, for FO and PRO, the solute flux is calculated independently of the solvent flux. In addition, the direction of the solvent flux differs between RO and the other two processes. Moreover, the PRO boundary condition includes the effect of transmembrane pressure, which is not present in the boundary condition for FO. Periodic and impermeable boundary conditions are not applicable to FO and PRO; hence, they are only employed in RO CFD simulations in which multi-scale modelling is later performed in order to estimate the performance of the whole membrane module. This is because in FO and PRO the conditions on both sides of the membrane are constantly changing. 

## 4. Hybrid RO Desalination Systems 

Although RO is the dominant technology for large-scale water desalination, the high energy requirement by the membrane component (of the order 2.2 kWh/m^3^), which accounts for more than half of the overall energy consumption (3.0–3.5 kWh/m^3^) is still the main concern for water operators [22]. Efforts to design energy-efficient RO membrane with comparable water flux and salt rejection have reached asymptotic trends [34,107] due to thermodynamic limits and the membrane permeability/selectivity trade-off [1,125]. In view of this, hybrid systems have been explored as alternative designs to address the limitations of conventional RO systems. Generally, membrane hybrid systems can be categorised into two types: (1) a combination of an RO membrane process with a traditional separation process (e.g., pervaporation and membrane distillation) [126] and (2) a combination of an RO membrane process with an emerging membrane process (e.g., FO and PRO) [22,127]. This section intends to focus on the latter type, with examples of hybrid system designs for desalination shown in Table 5.

**Figure 7:**
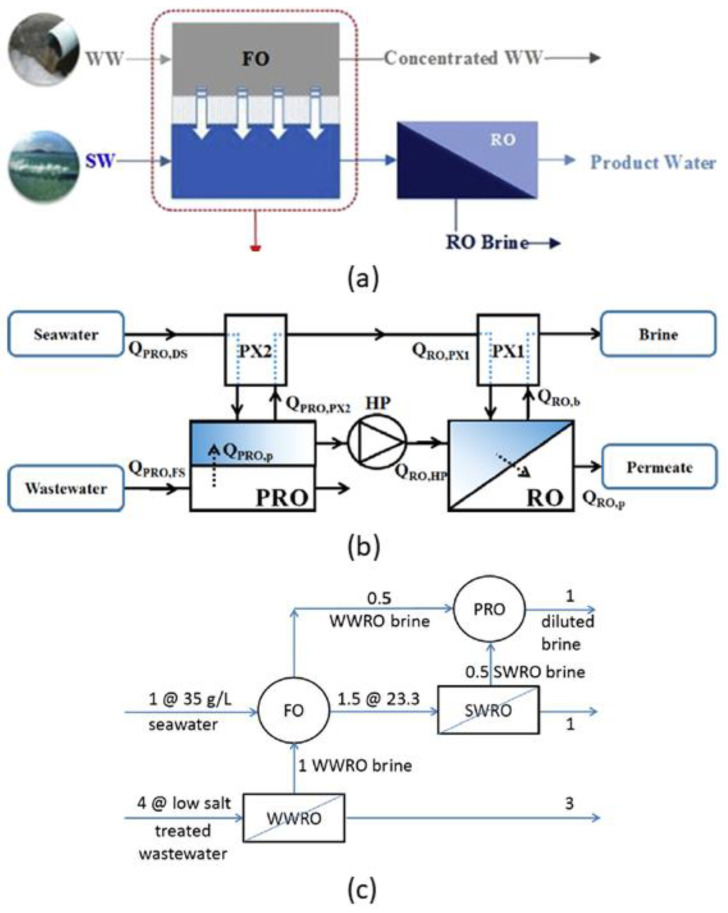
Examples of (**a**) FO–RO [132], (**b**) PRO–RO [14] and (**c**) FO–RO–PRO hybrid systems [22].

One of these hybrid options for RO desalination is the FO–RO hybrid system. These two processes are combined because the FO operation alone cannot achieve desalination by means of decreasing the concentration of the draw solution as a result of dilution by the water drawn from feed solution [128]. Therefore, the FO process requires additional operations to continuously or effectively regenerate the draw solution. In addition, the FO process in the FO–RO hybrid system can be beneficial in terms of freshwater recovery and enhanced pollutants removal (e.g., organic matter, scaling precursors and boron) [129,130]. It has been demonstrated that a FO hybrid process shows potential for desalinating water; for example, a FO–RO hybrid plant built in South Korea with a capacity of 2000 m^3^/day utilised an FO step to dilute seawater from an inlet solute mass fraction (*w_b,in_*) of 0.034 to a range of 0.010–0.013, reducing energy consumption by up to 25% compared with traditional SWRO [131]. Kim et al. [132] also found that the osmotic dilution of seawater through FO and/or a PAO pre-treatment process (Figure 7a) reduces the total specific energy consumption (SEC) by more than 55% compared to a simple RO. 

One major limitation of FO–RO hybrid systems, however, is the low water production in the FO pre-treatment [133,134]. Nevertheless, it must be pointed out that FO pre-treatment still shows advantages of lowering fouling propensity and removing dissolved constituents in feed water before they are fed into the RO process [134,135]. Besides the FO–RO hybrid system, a PRO pre-treatment process can also be used to reduce energy the consumption in the RO process. Fresh seawater feed is first diluted in a PRO operation, with wastewater as feed stream, before being sent to the RO stage. A major difference between FO and PRO pre-treatment is that the latter shows advantages in harvesting osmotic energy, thus further reducing energy consumption compared to FO pre-treatment. This agrees with the assessment by Kim et al. [14] (Figure 7b), who reported that a PRO–RO hybrid system could outperform the conventional RO system and the FO–RO process by lowering SEC by 51% and 16%, respectively. 

Senthil and Senthilmurugan [136] found that the post-treatment of RO brine using PRO, followed by recycling back to feed stream resulted in the lowest SEC among the different RO–PRO hybrid system configurations studied, which was around 49% lower compared with a conventional RO system. Fane [22] proposed a FO–RO–PRO hybrid system (Figure 7c) to reduce net energy required for water production. The numerical calculations involved three steps: (i) dilution of seawater in FO pre-treatment using wastewater RO reclamation brine as feed solution, (ii) RO process and (iii) PRO process using RO brine and wastewater brine from the FO step. It was suggested that each of the FO and PRO steps in the hybrid system could save up to 0.5 kWh/m^3^. All these findings reinforce that process intensification in which both membrane processes are combined into a single membrane unit has potential to further reduce cost compared with standalone RO. This opens more opportunities for finding the best process intensification with RO that could maximise water production performance with lowest possible energy consumption. The main challenges currently faced by hybrid RO desalination systems that increase energy consumption are fouling, internal and external concentration polarisation, all of which reduce solvent flux. Therefore, some strategies for dealing with these problems are the development of anti-fouling membranes [137,138,139], increased membrane permeance [107,140,141] and improved spacer design [52,55,56,57,58], particularly for the FO and PRO units.

## 5. Module Performance Metrics

### 5.1. Reverse Osmosis (RO)

An ideal SWM desalination system is the one which shows superior permeation flux, low propensity to fouling and low energy consumption. This highlights the need for critical criteria to assess the efficiency of a SWM desalination system from a selection of best spacer geometries, operating conditions and module configurations. It is worth noting that a number of module performance metrics are commonly used in the literature and, thus, this section intends to discuss their relevance in the context of membrane-based desalination.

High energy consumption remains an important issue that needs to be overcome for membrane-based desalination applications. Energy loss across the membrane is inevitable, and this represents the largest portion of energy losses in the membrane separation process. Karabelas et al. [105] suggested that energy consumption across the membrane (i.e., energy consumption to overcome osmotic pressure and membrane resistance) accounts for 77% and 72% of energy losses for seawater RO and brackish water RO, respectively. This suggests that most of the energy consumption mentioned earlier, of the order of 2.2 kWh/m^3^, is due to the pressure loss across the membrane, not along the channel. Furthermore, at a higher rate of recovery (that is higher flux), less energy can be recovered because the flow of retentate decreases. 

Given that energy consumption remains an important issue for RO, many energy performance metrics have been proposed. These include (1) specific power consumption (SPC) [50,51,54,142], (2) spacer configuration efficacy (SCE) [51,54,142], (3) spacer performance ratio (SPMP) [51,143] and (4) specific energy consumption (SEC) [104,105]. Of these, SEC is the most commonly cited indicator for measuring SWM performance. The main advantage of SEC is that it considers all the energy consumption contributions in the membrane unit (e.g., permeance) as well as the energy recovered through energy recovery devices (ERD), whereas other performance metrics such as SPC, SCE and SPMP only consider pressure loss along the membrane channel. 

While the above-mentioned parameters are useful to describe module performance, they do not explicitly address the economic impacts of energy consumption, especially for a large-scale system. Despite previous studies having evaluated the economic impacts [144,145,146] or total cost [147] of large-scale desalination systems, only a handful of studies have evaluated the impact of spacer design on total cost [148]. A recent techno-economic study by Toh et al. [149] found that improving spacer design is more crucial than increasing permeance at same recovery, especially for SWRO systems. In addition, the sensitivity analysis in that study reveals that the cost reduction associated with using advanced spacers for high-permeance membranes is independent of economic assumptions (e.g., amortisation factor and membrane cost). Thus, this suggests that sensitivity studies coupled to economic assessments can greatly assist in evaluating the factors affecting desalination cost.

It should be noted that economic analysis requires not only the operating conditions but also other information such as costs of electricity, equipment, labour and land [46]. Liang et al. [46] described a simplified economic model to evaluate an RO process with respect to the cost per unit volume of water treated. The main parameters involved in that analysis are the pre-treatment cost, the operating pressure, the pressure drop along the membrane channel and the membrane unit capital cost. The simplified economic assessment eliminates many complexities for the sake of facilitating the comparison of the effect of spacer design on the cost of desalination. In that sense, it does not evaluate the entire RO process, but rather the aspects affected by the spacer configuration.

The modelling of membrane processes can involve different ranges for both the spatial (length) and temporal scales of interest. In terms of length scales, membrane models can be categorised into “unit cell” (of the order of several millimetres) or module-scale (of the order of several meters). It is notable that attempting to resolve both the small and large spatial scales of a RO process simultaneously is nearly impossible, because it would require very large computational loads, making the model intractable, or significant simplifications that would trivialise the inclusion of the small-scale model. A 3D CFD model consisting of several unit cells or several millimetres in length, for instance, would require several tens of millions of discretised elements to resolve the local flow and mass transfer precisely [46]; hence, the detailed modelling of a full-scale module would be thousands of times that size. To overcome this challenge, the performance of module-scale RO can be evaluated using a multi-scale approach in which correlations for mass transfer and pressure drop obtained by data fitting the results of small-scale models are used [46,141]. With respect to temporal scales, models can be categorised into either steady (aiming to selecting best module design) or transient flow simulation (aimed at understanding how the solutes or fouling components interact with the membrane surface). For transient simulation, the time scale can be categorised into short and long time scales, where the short scale refers to the transient simulations that resolve individual vortices as they travel through the membrane channel and affect mass transfer. On the other hand, the long scale simulations aim at resolving the evolution of fouling (e.g., biofouling) require much longer time scales.

Nevertheless, understanding the interactions between the local spatial and temporal variations in flow and mass transfer fields is crucial in order to design a better SWM geometry. In view of this, this section first discusses the metrics that can be obtained from CFD simulation to measure the local performance at the unit cell scale. Generally, CFD simulations can provide data such as the local flow velocity, mass transfer coefficient (*k_mt_*), water flux (*J_w_*), solute concentration distribution (e.g., CP modulus) as well as the pressure drop (or Fanning friction factor, *f_glob_*). These parameters are useful in visualising the local hydrodynamics and mass transfer performance. By analysing flow velocity and solute profiles in 2D unsteady CFD simulations, Fimbres Weihs et al. [150] found that there are two main mechanisms responsible for mass transfer enhancement, namely increased wall shear and flow of lower concentration fluid into the concentration boundary layer. In terms of measuring vortex strength, the lambda-2 (*λ_2_*) criterion from CFD can be used to identify vortices that are responsible for pressure loss and fluid mixing [74]. 

Despite the availability of performance metrics for RO, most of these are only applicable for steady state operating conditions, without taking into consideration the effects of membrane fouling. It must be emphasised that membrane fouling tends to have a significant impact on the SEC and total processing cost over long periods of operation, and future work on the analysis of this issue are therefore highly recommended. 

### 5.2. Forward Osmosis (FO) and Pressure Retarded Osmosis (PRO) 

The main parameters affecting the FO process are solute concentration in both draw and feed solution, reverse draw solute flux [6,151] and membrane salt rejection [112]. In terms of hydrodynamics analysis, several studies [109,110] introduced the structural parameter (*S*), which is used to evaluate effect of the porous layer geometry on ICP:

(2) $$S=\frac{t_{s}T}{\varepsilon }$$

where *t_s_*, T and *ε* represent the support layer membrane thickness, diffusive tortuosity and porosity, respectively. The *S* parameter is also known as the intrinsic structural parameter (*S_int_*) for FO membranes. The effective structural parameter (*S_eff_*) [110], on the other hand, is an alternative calculation of the *S* parameter using experimental measurements, due to difficulties in measuring diffusive tortuosity (T). Despite this, it is found that inconsistencies exist between *S_int_* and *S_eff_*. Kang et al. [110] explained that the inconsistencies are due to the misinterpretation of interfacial transport phenomena between the active layer and the support layer. They therefore proposed a modified ${\tilde{S}}_{eff}$ parameter to increase the accuracy of measurements. In a separate work, Lee et al. [109] also found that the modified ${\tilde{S}}_{eff}$ parameter may show small discrepancies with the *S_int_* for non-straight support layer pore geometries due to tortuosity, but still sufficient to evaluate the impact of surface porosity on the FO process. 

PRO, on the other hand, plays an important role as an energy generating operation, and is usually assessed in terms of permeate flux and density of energy production. Energy generation can be evaluated in terms of power density (*PD*) [12,43,120,152]:

(3) $$PD=J_{w}\Delta p$$

where *J_w_* and Δ*p* denote transmembrane water flux and transmembrane hydraulic pressure difference, respectively. The power density is the main interest of PRO process and is usually measured in the form of average power density and specific energy. The average power density ($\bar{PD}$) considers the power density variation along the module of a full-scale PRO system [120]:

(4) $$\bar{PD}=\frac{\Delta p\Delta Q}{A_{m}}$$

whereas specific energy (*SE*) elaborates on the energy extracted per unit of volumetric flow rate in both the feed and the draw [120]:

(5) $$SE=\frac{\Delta pQ_{p}}{Q_{f,in}+Q_{d,in}}$$

A study conducted by Soltani and Struchtrup [121] used these same definitions (for $\bar{PD}$ and SE), but they replaced the term ΔpΔQ with *W_net_*, which is the system output that considers both power consumption (to pressurise the stream in a pump) and generation (obtained from depressurising in the turbine). 

Table 6 summarises the performance metrics typically employed in the small- and large-scale analysis of osmotic membrane processes. Of these indicators, SEC is perhaps the most commonly cited indicator that is used to compare different osmotic membrane processes. However, particular attention should be given when using this metric because the resulting SEC can vary depending on inlet concentration, recovery, pump efficiency and model equations employed [141]. It must be pointed out that no performance metric can be considered universal for comparing all osmotic membrane processes. 

### 5.3. CFD-Derived Metrics

Performance metrics used for evaluating membrane processes at the small-scale can also be used to understand the flow field, which is important for optimising large-scale osmotic membrane processes. For RO process, the competing effect between mass transfer rate (e.g., Sherwood number, *Sh*) and power loss (e.g., Fanning friction factor, *f_glob_* or power number, *Pn*) is the most representing metric for this type of membrane unit evaluation. Several studies have observed that larger wall shear is generally correlated with larger mass transfer [150]. Moreover, maximum wall shear stress (*τ_t_*) can be used as a proxy indicator for fouling reduction, given that a greater shear rate has a potential to minimise fouling. Pressure loss metrics in the FO process, on the other hand, are not that relevant. Instead, mass transfer related parameters such as *Sh* and CP are typically used as performance metrics for this membrane process. For PRO, the most important metrics are *Sh* and mass transfer coefficient (*k_mt_*) which are directly related to *PD*.

When considering hybrid RO desalination systems, an important value to use as a reference is the Gibbs free energy of mixing of the salt/water solution (∆*G_mix_*), typically expressed in kWh/m^3^ [22]. This value can be considered the reversible energy of mixing, thus establishing the thermodynamic limit on the minimum amount of energy required to carry out the separation of the dissolved salts and the water. Therefore, a simple indicator of the efficiency (in % terms) of a hybrid RO desalination system is the ratio of specific energy consumption (*SEC*) to ∆*G_mix_*. For seawater, the value of ∆*G_mix_* is around 1 kWh/m^3^, but current typical RO systems require more than twice this amount to desalinate seawater [22], thereby resulting in an energy efficiency below 50%. However, these energy requirement values refer to the complete desalination plant, and not just to the RO step that, although it is the largest energy consuming operation in a desalination plant, is not by any means the only one. Furthermore, the use of energy recovery devices (ERDs) to reduce the high-pressure pumping power requirement reduces the overall energy consumption of the plant, and this generally outside the scope of a CFD simulation. For the FO unit, as there is no energy input, energy efficiency cannot be defined in a similar manner; however, FO can help reduce the energy required in a desalination plant. For PRO, energy is also recovered outside the PRO unit itself, by means of a turbine or an ERD, so the efficiency of the system is impacted by the specific energy recovery system and not just the membrane unit.

From the mass transfer point of view, an efficiency can also be defined in terms of the ratio of expected flux without any concentration polarisation or fouling, compared to the flux predicted by CFD. This efficiency would be impacted by the operating conditions, such as fluid regime, transmembrane pressure, salinity and spacer configuration, so it is a simple metric that can be readily determined from CFD simulations. In addition, our previous work proposed a metric for measuring effectiveness of hydrodynamic perturbation techniques (e.g., electro-osmotic forced slip) in terms of relative change in CP index:

(6) $$\Phi =1-\frac{\gamma_{E}}{\gamma_{NE}}$$

where *E* and *NE* identify the CP indices for the enhanced and non-enhanced conditions. A positive value of Φ indicates an effective mass transfer enhancement whereas a negative value of Φ signals a decrease in mass transfer, and hence flux [158].

## 6. Conclusions

This article provides an overview of the recent developments of the modelling of different osmotic membrane processes (i.e., RO, FO and PRO) and summarises the different indicators used to evaluate the performance and efficiency of those membrane processes. Optimising the spacer geometry in SWM modules remains an important topic, especially for RO, and this review concludes that the CFD modelling of complex spacer geometries is still limited to hydrodynamics (without mass transfer), particularly due to the difficulties associated with generating high quality discretisation meshing near the intricate features of complex spacers. Thus, there are many research opportunities for understanding the interactions between mass transfer and foulants using complex spacer geometries. 

From the multi-scale modelling perspective, no detailed full models that consider fouling effects are yet to be found in the literature. Current multi-scale models are only capable of predicting the early stages of operation of osmotic membrane processes, prior to the onset of fouling. Thus, future work that considers fouling is highly recommended in order to improve predictive capabilities and further our understanding of the intricate mechanisms and processes that affect membrane performance under real conditions.

While existing CFD models for FO membrane processes have been validated against experimental work, such research however is still rare for PRO membrane processes. Future studies utilising CFD models should address validation for PRO. One recommendation is to employ PIV technology to obtain information adjacent to the membrane surface and compare it with CFD data. 

With respect to the assessment of membrane module performance, it is highly unlikely that it will be possible to use a single metric to describe the overall performance of any osmotic membrane processes. Nevertheless, sensitivity studies coupled to economic assessments may be able to shed enough insights into what the most important design characteristics are for minimising the cost of desalination.

## Figures and Tables

**Table 1:** Summary of novel spacer designs developed.

Authors	Research Type	Geometry Analysed	Main Findings	Observations
Koutsou and Karabelas [56]	CFD and experimental	Submerged spacer with circular node with varying mesh length and attack angle.	Novel spacers show 15% better performance in terms of SCE compared with conventional spacers. Novel spacers show potential for lower pressure loss compared to conventional spacer under high Reynolds number (*Re_ch_* ≈ 200).	No detail on meshing was provided.
Han et al. [66]	CFD	Net type symmetric spacer with different diameter and pillar shape nodes.	Higher porosity results in lower pressure drop and wall shear stress. Proposed a modified friction factor to calculate area average shear stress.	Mass transfer phenomena were not studied.
Kerdi et al. [55]	CFD and experimental	Effect of spacer perforation on hydrodynamics.	Perforations introduce jet-effect, thus eliminate recirculation zones and hamper growth of fouling. 1-hole spacer results in greatest flux enhancement of 75% with pressure drop reduction of 15%. OCT scans show that 1-hole spacer exhibits the lowest fouling degree. 3-hole spacer results in the highest pressure drop reduction of 54% with 17% flux enhancement.	The 3D unsteady CFD is only limited to hydrodynamics, mass transfer not included.
Sreedhar et al. [52,63]	Experimental	3 TPMS-based spacers for UF and RO processes.	All TPMS spacers improve flux (15.5%), reduce pressure drop (5–12.5%).	No CFD modelling of mass transfer was conducted, hence no details on the local flow and mass transfer. Only applicable to lab scale and questionable implementation for large scale SWM module due to sharp edges of spacer.
	Experimental	6 TPMS spacers for UF, and the effect of grading spacer voidage on one of TPMS spacers.	All TPMS spacers show mass transfer enhancement and lower CP. Change of TPMS spacer directionality has significant impact on system performance. Functionally graded spacer porosity does not improve system performance. Higher spacer voidage improves mass transfer, reduces pressure loss and manufacturing materials.	
Ali et al. [58]	CFD and experimental	Net-type symmetric spacer with small diameter (*d_f_*/*h_ch_* = 0.42) and column shape nodes.	CFD simulation shows that proposed spacer reduced pressure drop by 3×. Proposed spacer is able to reduce fouling and SEC by 79% and 50%, respectively. Besides, it improves average flux by 100%.	The base case of comparisons is not a typical or conventional spacer geometry (i.e., dual-layer spacer geometry).

**Table 2:** Recent numerical studies and computational fluid dynamics (CFD) modelling of the FO process.

Researchers	Significance	Findings	Observation
Tan and Ng [114]	Studied the effect of both ICP and ECP on flux.	The proposed model showed more accurate prediction on water flux if both ICP and ECP phenomena are modelled.	The study is restricted to 1D study.
Gruber et al. [116]	Development of CFD model based on semi-analytical models.	The effect of ECP on the porous support cannot be neglected. The proposed model using open source CFD code efficiently run steady and unsteady simulations at low computational cost	Pioneering CFD study for FO process.
Park and Kim [41]	Propose concentration polarisation index (CPI) as CP indicator.	Unlike RO process, boundary layer compression is more effective in mitigating CP for FO process compared to boundary layer disruption.	Spacer study was limited to 2D analysis.
Sagiv et al. [115]	Comparison of ICP and ECP obtained from experiment, CFD and film model analysis.	Film model approximation overestimated the permeation resistance of membrane support layer compared with CFD analysis.	ECP on membrane feed side was not considered.
Heon et al. [111]	Development of full-scale FO SWM model.	Use of fitting process on several parameters (feed solution flow, draw solution flow and transmembrane pressure) to enhance module accuracy.	Data obtained from experimental work was required.
Lee et al. [109]	Development of pore-scale CFD simulator to study effect of surface porosity, bulk porosity and pore geometry.	Increase in bulk porosity reduces ICP and increase effective osmotic pressure. Increase in surface porosity increases water flux.	Oversimplified support layer pore geometry may not represent real membrane.
Lian et al. [36]	3D CFD modelling for FO and PAO process in both spiral wound and plate and frame configurations.	Increase in feed side inlet pressure induces membrane displacement in PAO mode, which reduces permeation flux especially for plate and frame module. Pressure drop in spiral wound module is more sensitive to cross flow velocity compared to that in plate and frame module.	CFD model cannot predict flux and pressure drop for high saline draw solution.
Alshwairekh et al. [119]	Development of 3D CFD model for FO process under both laminar and turbulent conditions.	Membrane corrugation enhanced mixing and alleviated ECP. Corrugation on both sides of membrane resulted in the highest flux.	Questionable modelling within turbulent flow as flow inside FO is typically laminar
Qing et al. [6]	Modelling of FO flow path in SWM module (U-shape path) against flat sheet membrane (I-shape path).	U-shape flow path resulted in large spatial flow variation but this did not really affect overall flux.	No spacer was considered in CFD modelling.

**Table 3:** Recent numerical study and CFD modelling for PRO process.

Researchers	Significance	Findings	Observation
Nagy [122]	Development of 1D PRO model.	Assumption of negligible feed side mass transfer resistance may overestimate membrane performance by 4–8%.	Early numerical study of PRO process.
Straub et al. [120]	Development of 1D module-scale PRO model.	Major challenge in full-scale PRO system is the power density and energy trade-off.	The study only considered river water and seawater pairing, which resulted in comparably low energy generation.
Wang et al. [43]	Development of CFD modelling for PRO process. Comparison between CFD modelling and semi-analytical model.	CFD modelling outperformed semi-analytic model in elucidating the effect of membrane properties.	Early CFD study of PRO process.
Sagiv et al. [123]	Development of a 2D finite element PRO model.	High efficiencies of pumps and energy recovery equipment are required together with high salinity differences in order to achieve greater net power generation. Higher permeability membranes could enhance PRO performance.	The simulation was limited to 2D analysis.
Soltani and Struchtrup [121]	Evaluation of large-scale PRO process.	Dual-stage PRO system results in lower SEC compared with single-stage system.	Effect of ECP not considered in the study.

**Table 4:** CFD boundary conditions for membrane channels in RO, FO and PRO processes [43,46,74,116,119,124].

Location	RO	FO	PRO
Inlet	$u=u_{in}\left(y,z\right)$ $u_{y}=0$ $u_{z}=0$ $w=w_{in}$		
Outlet	$\frac{\partial u_{x}}{\partial x}=\frac{\partial u_{y}}{\partial x}=\frac{\partial u_{z}}{\partial x}=\frac{\partial w}{\partial x}=0$		
Wall (non-membrane, spacer)	$u_{x}=u_{y}=u_{z}=\frac{\partial w}{\partial n}=0$		
Side openings	${\vec{v}}_{A}={\vec{v}}_{B}$ $p_{A}=p_{B}$ $w_{A}=w_{B}$ ${\left(\nabla \vec{v}\right)}_{A}={\left(\nabla \vec{v}\right)}_{B}$ ${\left(\nabla p\right)}_{A}={\left(\nabla p\right)}_{B}$ ${\left(\nabla w\right)}_{A}={\left(\nabla w\right)}_{B}$		
Inlet/Outlet (periodic)	${\vec{v}}_{in}={\vec{v}}_{out}$ ${\left(\nabla \vec{v}\right)}_{in}={\left(\nabla \vec{v}\right)}_{out}$ ${\left(\nabla p\right)}_{in}={\left(\nabla p\right)}_{out}$ ${\left(\frac{w_{m}-w}{w_{m}-w_{b}}\right)}_{in}={\left(\frac{w_{m}-w}{w_{m}-w_{b}}\right)}_{out}$	N/A	N/A
Membrane permeation	$u_{x}=u_{z}=0$ $u_{y}=A\left(\Delta p-\sigma \phi Rw_{m}\right)$ $u_{y}w_{m}R-D{\left(\frac{\partial w}{\partial y}\right)}_{m}=0$	$u_{x}=u_{z}=0$ Selective layer: $u_{y}=A\Delta \pi$ Porous layers: $u_{y,m}\frac{\partial w}{\partial y}=D\frac{\partial^{2}w}{\partial y^{2}}$ Whole membrane: $u_{y}=\frac{1}{K}\ln\left(\frac{B+A\pi_{d}}{B+\left|J_{w}\right|+A\pi_{f}}\right)$ $u_{y}w_{m}-D{\left(\frac{\partial w}{\partial n}\right)}_{m}=\frac{J_{s}}{\rho }$ $J_{s}=-\frac{B}{\phi \cdot A}J_{w}$	$u_{x}=u_{z}=0$ Feed side: $u_{y,f}=A\left[\sigma \phi \left(w_{d,m}-w_{f,m}\right)-\Delta p\right]$ $u_{y,f}w_{f,m}-D{\left(\frac{\partial w}{\partial y}\right)}_{f,m}=\frac{B}{\rho_{f}}\left(w_{d,m}-w_{f,m}\right)$ Draw side: $u_{y,d}=u_{y,f}\frac{\rho_{f}}{\rho_{d}}$ $u_{y,d}w_{d,m}-D{\left(\frac{\partial w}{\partial y}\right)}_{d,m}=\frac{B}{\rho_{d}}\left(w_{d,m}-w_{f,m}\right)$
Impermeable membrane	$u_{y}=0$ $w=w_{m}$ *J_w_* calculated using *k_mt_*	N/A	N/A

**Table 5:** Current proposed hybrid systems for RO desalination.

Hybrid System	Reference	Main Findings	Obstacles
FO–RO hybrid system	Kim et al. [132]	PAO pre-treatment reduces SEC of RO process by more than 55%.	Requirement to maintain higher feed pressure than draw pressure throughout membrane module.
PRO–RO hybrid system	Kim et al. [14]	PRO–RO hybrid system reduces SEC by 51% and 16% compared with standalone RO and FO–RO hybrid system, respectively.	Susceptibility of PRO to membrane fouling may cause flux decline and thus require anti-scaling pre-treatment.
RO–PRO hybrid system	Senthil and Senthilmurugan [136]	RO–PRO hybrid system reduces SEC up to 49% compared to standard RO system.	The calculation does not consider possible flux decline due to fouling.
FO–RO–PRO hybrid system	Fane [22]	FO or PRO hybrid process shows potential energy benefit of 0.5 kWh/m^3^.	Main challenge due to operation and capital costs.

**Table 6:** Summary on important indicator metrics used for membrane processes.

Indicator Metric	Mathematical Description	Unit	Description	Observation
Concentration polarisation [153]	$\gamma =\frac{w_{w}}{w_{b,in}}$	-	Ratio of solute concentration at the membrane wall to the concentration at the inlet bulk.	The formulation of CP index varies depending on the choice of membrane processes used (viz. RO vs FO).
Ideal energy efficiency of desalination [22]	$\eta_{id}=\frac{\Delta G_{mix}}{SEC}$	-	Ratio of Gibbs free energy of mixing of salt/water mixture versus specific energy consumption for desalination.	Current technologies have efficiencies just below 50% of the ideal thermodynamic limit.
Fanning friction factor [154]	$f_{glob}=\frac{d_{h}}{2\rho u_{eff}^{2}}\frac{\Delta p_{ch}}{L}$	-	Dimensionless measurement of pressure loss across the membrane channel.	Only related to hydrodynamics, not mass transfer.
Local mixing index [155,156]	$M_{loc}={\left(\frac{\partial u_{p}}{\partial x}\right)}^{2}+{\left(\frac{\partial u_{p}}{\partial y}\right)}^{2}+{\left(\frac{\partial v_{p}}{\partial x}\right)}^{2}+{\left(\frac{\partial v_{p}}{\partial y}\right)}^{2}$	s^−2^	Mixing dependence on fluid stretching and folding.	The relationship between the degree of stretching and folding measured, and mass transfer enhancement is unclear.
Mass transfer coefficient [157]	$k_{mt}=\frac{D}{w_{w}-w_{b}}{\left(\frac{\partial w}{\partial y}\right)}_{w}$	m/s	Diffusion rate constant of water through membrane wall.	Give quick prediction of the degree of mass transfer enhancement. Typically, the values are of the order of 10^−^^5^ m/s.
Mass transfer enhancement factor [158]	$\Phi =1-\frac{\gamma_{E}}{\gamma_{NE}}$	-	Relative change in concentration polarisation due to enhancement techniques.	Positive values indicate increased mass transfer, whereas negative values indicate a decrease in mass transfer and flux.
Power density [120]	$PD=J_{w}\Delta p$	W/m^2^	Power generated per membrane area (for PRO application).	High power density is favourable to minimize the membrane area required for generating power.
Recovery rate [46]	$R_{r}=\frac{Q_{p}}{Q_{in}}$	-	Ratio of volumetric permeate to feed flow rate.	Provides quick prediction of total water produced depending on types of feed water used (viz. brackish vs seawater).
Sherwood number [159]	$Sh=\frac{k_{mt}d_{h}}{D}$	-	Ratio of mass transfer by convection to mass transfer by diffusion.	Only diffusivity term that reflects the solute characteristics and does not take into consideration other membrane properties parameters (e.g., surface charge).
Spacer configuration efficacy [142]	$SCE=\frac{Sh}{Pn}$	-	Ratio of mass transfer increment by spacer filaments to power consumption.	The limitation of this concept is that it does not show much dependence on Reynolds number.
Specific energy consumption [29]	$SEC=\frac{W_{TOTAL}}{Q_{p}}$	kWh/m^3^	Ratio of energy consumption to volumetric permeate flow rate.	Most commonly used for predicting energy usage but does not reflect the actual processing cost.
Wall shear stress [160]	$\tau_{t}=\sqrt{\tau_{x}^{2}+\tau_{y}^{2}+\tau_{z}^{2}-{\left(\mu \frac{\partial \vec{v}}{\partial n}\cdot \hat{n}\right)}^{2}}$	Pa	Rate of change of velocity near the membrane surface.	Proxy indicator for anti-fouling tendencies.

## Nomenclature

*Symbol*	
*A*	Membrane permeability [m Pa^−^^1^ s^−^^1^]
*B*	Solute permeability [m s^−^^1^]
*D*	Solute diffusivity [m^2^ s^−^^1^]
*d_h_*	Hydraulic diameter [m]
*f_glob_*	Fanning friction factor
*h_ch_*	Membrane channel height [m]
*K*	Diffusion resistivity within the membrane porous layer [s m^−^^1^]
*k_mt_*	Mass transfer coefficient [m s^−^^1^]
*J_w_*	Transmembrane volumetric water flux [m s^−^^1^]
*J_s_*	Solute mass flux [kg m^−^^2^ s^−^^1^]
*L*	Membrane channel length [m]
*M_loc_*	Local mixing index [s^−o^]
*n*	Membrane surface normal vector
*Q*	Volumetric flow rate [m^3^ s^−^^1^]
*Q_p_*	Permeate flow rate across membrane [m^3^ s^−^^1^]
*p*	Pressure [Pa]
Δ*p*	Transmembrane hydraulic pressure difference [Pa]
Δ*p_ch_*	Channel pressure drop [Pa]
PD	Power density [W m^−^^2^]
*Pn*	Power number
*R*	Membrane intrinsic rejection
*R_r_*	Recovery rate
*S*	Structural parameter [m]
SE	Specific energy [J m^−^^3^]
SEC	Specific energy consumption [J m^−^^3^]
*Sh*	Sherwood number
SCE	Spacer configuration efficacy
SPC	Specific power consumption [W m^−^^3^]
SPMP	Spacer performance ratio
*t*	Membrane thickness [m]
*u_eff_*	Effective velocity [m s^−^^1^]
*u_x_*	Flow velocity in *x*-direction [m s^−^^1^]
*u_y_*	Flow velocity in *y*-direction [m s^−^^1^]
*u_z_*	Flow velocity in *z*-direction [m s^−^^1^]
$\vec{v}$	Velocity vector [m s^−1^]
*v_p_*	Perturbation velocity vector [m s^−^^1^]
*w*	Solute mass fraction
*x*	Distance in the bulk flow direction, parallel to membrane surface [m]
*y*	Distance from the bottom membrane surface, in direction normal to the surface [m]
*z*	Distance in the direction perpendicular to both *x* and *y* [m]
*Greek letters*	
*ε*	Porosity
*γ*	Concentration polarisation modulus
*η_id_*	Ideal energy efficiency of desalination
*μ*	Dynamic viscosity [kg m^−1^ s^−1^]
*π*	Osmotic pressure [Pa]
*ϕ*	Osmotic pressure coefficient [Pa]
Φ	Mass transfer enhancement factor
*ρ*	Fluid density [kg m^−3^]
*σ*	Reflection coefficient
T	Tortuosity
*τ*	Wall shear stress [Pa]
*Subscript*	
*b*	Value for the bulk flow
*d*	Value for the draw solution
*E*	Value for enhanced condition
*f*	Value for the feed solution
*in*	Value at the domain inlet
*out*	Value at the domain outlet
*m*	Value at membrane layer/surface
*NE*	Value for non-enhanced condition
*p*	Value for the permeate
*s*	Value for the support substrate
*slit*	Value in empty channel
*spacer*	Value in spacer-filled channel
*t*	Value for wall-tangential shear
*w*	Value on the membrane surface (wall)
